# Factors Affecting Women’s Attitudes Toward the Use of Epidural Anesthesia During Labor in Riyadh in 2023

**DOI:** 10.7759/cureus.47268

**Published:** 2023-10-18

**Authors:** Ahmed A Alrizqi, Mona A Alrizqi, Abdulaziz A Alrizqi, Leen Alsabti, Rawan A Alsomali, Abdulrahman Hussamuldin

**Affiliations:** 1 Radiology, King Saud Medical City, Riyadh, SAU; 2 College of Medicine, Al Maarefa University, Riyadh, SAU; 3 Medical School, Al Maarefa University, Riyadh, SAU; 4 Medicine and Surgery, Al Maarefa University, Riyadh, SAU

**Keywords:** ea (epidural anesthesia), pregnancy, pregnant, saudi women, labor

## Abstract

Background

Epidural analgesia (EA) is a type of central nerve block achieved by injecting a local anesthetic near the pain-transmitting nerves. It is commonly used to relieve pain during labor. The intensity of pain experienced and the effectiveness of pain relief can affect a woman’s labor and delivery experience. Labor pain is a long-lasting and debilitating pain that women experience; therefore, pain relief techniques have become widely available. However, not all women are willing to use EA for pain relief. The factors that influence women’s choices regarding EA should be explored. This study aimed to comprehend women’s perspectives on EA in Riyadh and clarify the variables influencing their decision-making.

Methodology

A total of 336 women in their reproductive years participated in this study. An online questionnaire with five sections was used to collect data. The socio-demographic information in the first section covered age, level of education, occupation, income, marital status, pregnancy, maternity, and prenatal care. The second section focused on how painful labor was for women who had already given birth. The third and fourth sections discussed labor pain relief techniques, including epidural anesthesia. The final section assessed the participant’s interest in receiving EA during the next delivery. Socio-demographic data were considered to be a predictor, whereas awareness levels and a desire to receive EA were seen as results. The information was cleaned, coded, and entered into SPSS version 29 (IBM Corp., Armonk, NY, USA). The results are presented as frequencies and percentages. A Likert scale was used for data analysis. Statistical significance was established at p-values <0.05.

Results

In total, 336 Saudi women were included in this study, 86% of whom were between the ages of 30 and 35 years, and 69% of whom had a university education. One-third of the women (32%) had a monthly income of 10,000 to 20,000 SAR, and 55% of them had experienced three or more pregnancies. Fear of pain was the main reason for using epidural anesthesia in more than half of the participants given the choice. The most reinforcing factor was obtaining proper and sufficient information about EA, while the most restrictive factor was the fear of harming the infant. In particular, women aged 30-35 years (i.e., most of the included women) were commonly influenced by negative factors. Marital status and educational level played no significant role in women’s use of EA.

Conclusions

Saudi women showed a good attitude toward EA. However, the awareness of EA was quite low. Fear of labor pain appeared to be the main reason for using EA. Health education programs on EA can increase the knowledge of and intent to use EA among women.

## Introduction

The amount of pain experienced and the effectiveness of pain relief may affect the labor and delivery experience and have immediate and long-term emotional and psychological effects. Epidural labor analgesia is a central nerve block achieved by the injection of a local anesthetic close to the nerves that transmit pain [1]. Labor pain is a common and severe form of suffering that women experience. It causes an unpleasant experience for women during labor [2]. Over the past 50 years, obstetric anesthesiology, a major subspecialty of anesthesia globally, has substantially advanced. The Society for Obstetric Anesthesia and Perinatology was established in 1968. Approximately 82.4% of all parturient women received some type of anesthetic or analgesic for cesarean section or labor, according to the SCORE (Serious Complication Repository) project on the use of obstetric anesthesia. The most popular type of labor analgesia (65%) is epidural analgesia (EA), followed by combined spinal EA [3]. Labor pain is continuous and debilitating; therefore, pain relief techniques have become widely available to alleviate it [2]. EA has become a popular and efficient form of labor pain management [1]. However, not all women are willing to receive EA for pain relief [1]. Therefore, it is essential to investigate the relevant factors that influence their decision regarding EA. Preferences for and awareness of EA among women have been investigated in several international studies. These studies have demonstrated the influence of culture and other factors on women’s perceptions of EA. A study in Ethiopia revealed poor practice of labor pain management methods due to a lack of awareness and education [3]. However, in Japan, EA is the most common form of labor analgesia [4]. To our knowledge, approximately eight studies on this topic have been conducted in Saudi Arabia. In 2022, a study in the eastern region of Saudi Arabia revealed that most participants decided not to request EA upon their next delivery [5]. Moreover, a 2023 study in Jazan revealed that women of childbearing age knew little about EA [6]. Likewise, a 2022 study in Jeddah involving 105 participants in the King Abdulaziz Medical City revealed a lack of awareness of EA among pregnant women [7]. Awareness levels differ in the vast territories of Saudi Arabia. Therefore, the main goal of this study was to comprehend women’s perspectives and clarify the elements influencing their decision-making on EA in Riyadh, the capital city of Saudi Arabia and the largest city on the Arabian Peninsula.

## Materials and methods

Study design and participants

This cross-sectional study involved Saudi women of childbearing age who were 18 years and above and lived in Riyadh. We excluded non-Saudi women, women living in other regions, and those less than 18 years old.

Study tool

A self-administered online questionnaire obtained from studies by Alshamri et al. [2] and Al Mousa et al. [5], with some modifications, was conducted in Riyadh between April and June 2023 at social gatherings. The questionnaire took approximately two minutes to complete. It was divided into five sections created in English, translated into Arabic, and reviewed by a team of specialists to resolve any inconsistencies. The first section collected socio-demographic data, including prenatal care, pregnancy, maternity, age, educational background, occupation, income, and marital status. The second section focused on how painful labor was for women who had already given birth. The knowledge of EA and labor pain relief techniques was covered in detail in the third and fourth sections. The final section assessed the participant’s interest in receiving EA during the next delivery. Socio-demographic information was considered a predictor, while awareness levels and desire to receive EA were regarded as results.

Sample size and ethical considerations

The study included 336 women with a confidence level of 95% and a 5% margin of error. Our Institutional Review Board approved this study at the College of Medicine, AlMaarefa University, Riyadh, Kingdom of Saudi Arabia (approval number: IRB23-059). All participants provided informed consent for the use of their information, and the safeguarding of their privacy was ensured. Moral considerations, cultural standards, and legal concerns were taken into account.

Statistical analysis

SPSS version 29 (IBM Corp., Armonk, NY, USA) was used to clean, code, and enter the data. The results were represented as frequency and percentage. Statistical significance was set at a p-value <0.05. The participants’ ratings of each component of the various outcome variables were used to calculate the satisfaction score. The average value was calculated using a Likert scale ranging from 1 to 5. A satisfaction score of 15 was used to determine the levels of dissatisfaction (below the mean) and satisfaction (equal to or above the mean) [8].

## Results

A total of 336 Saudi women participated in this study. Within this group, 86% were between 30 and 35 years old, 68.5% had received a high school education, 32% enjoyed a well-established monthly income of approximately 10,000 SAR to 20,000 SAR, and 55% had undergone three or more pregnancies. Our study included four age groups, i.e., 40 and above (31%), 30-35 (32%), 25-29 (31%), and 18-24 years (9%). However, negative factors had a greater influence on the two higher age groups, particularly those aged 30-35 years. Women in their fourth decade were highly impacted by the negative factors rather than the positive ones (Table 1).

**Table 1:** Socio-demographic data (N = 336).

	Frequency	Percent
Age (years)		
18–24	31	9.2
25–29	93	27.7
30–35	108	32.1
40 and above	104	31.0
Education level		
Illiterate	6	1.8
Primary school	6	1.8
Middle school	94	28.0
High school	230	68.5
University degree	0	0.0
Monthly salary		
Less than 5,000	93	27.7
5,000 to 10,000	106	31.5
1,000 to 20,000	108	32.1
More than 20,000	29	8.6
Pregnancies		
One	68	20.2
Two	84	25.0
Three and more	184	54.8
Social status		
Married	288	85.7
Divorced	36	10.7
Widowed	12	3.6
Total	336	100.0

Previous exposure to epidural anesthesia was reported by 79% of the participants. The awareness of epidural anesthesia varied among women, yet 51% of them had learned about it from family and friends (Table 2).

**Table 2:** Previous experience and source of knowledge (N = 336).

Question	Frequency	Percent
Have you received an epidural injection before?		
Yes	266	79.2
No	70	20.8
How did you get to know about epidural injection?		
Family and friends	172	51.2
Doctors	116	34.5
Social media	48	14.3
Total	336	100.0

Further, 58% of the participants reported that fear of pain was the primary reason for seeking EA. Regarding factors that reinforce women’s decision toward using EA during childbirth, 44% considered a well-structured educational program about EA to be important, while 30% emphasized the significance of a good support system, and 42% highlighted the impact of positive experiences shared by family and friends. These aspects strongly influenced their decision-making. Conversely, the factors that adversely influenced women’s decisions to accept EA during childbirth were concerns about affecting the infant during labor (40%), medical issues preventing the use of EA (40%), religious or cultural considerations (35%), previous negative experiences with EA (33%), and apprehensions about impacting the labor outcomes (34%). Approximately 28% of respondents were uncertain, and 34% agreed to some extent with avoiding epidural anesthesia due to its complications (Table 3).

**Table 3:** Factors affecting the decision-making (N = 336).

Questions	Frequency	Percent
Do you think having enough information or education on epidural injections contributed to your decision?		
Strongly disagree	15	4.5
Disagree	42	12.5
Agree to some extent	70	20.8
Agree	61	18.2
Strongly agree	148	44.0
Did support from your family or husband influence your decision?		
Strongly disagree	27	8.0
Disagree	82	24.4
Agree to some extent	62	18.5
Agree	65	19.3
Strongly agree	100	29.8
Did the positive experiences of people around you influence your decision?		
Strongly disagree	12	3.6
Disagree	44	13.1
Agree to some extent	55	16.4
Agree	84	25.0
Strongly agree	141	42.0
Do you think that receiving an epidural injection affects the nature of childbirth?		
Strongly agree	74	22.0
Agree	80	23.8
Agree to some extent	95	28.3
Disagree	80	23.8
Strongly disagree	7	2.1
Do you think that an epidural injection affects the health of the fetus?		
Strongly agree	43	12.8
Agree	56	16.7
Agree to some extent	75	22.3
Disagree	136	40.5
Strongly disagree	26	7.7
Did you have any health factors that prevented you from receiving an epidural injection?		
Strongly agree	25	7.4
Agree	18	5.4
Agree to some extent	29	8.6
Disagree	133	39.6
Strongly disagree	131	39.0
Do you believe that epidural injections interfere with the natural course of childbirth according to religion and culture?		
Strongly agree	41	12.2
Agree	21	6.3
Agree to some extent	48	14.3
Disagree	118	35.1
Strongly disagree	108	32.1
Do you think that epidural injections cause complications after childbirth?		
Strongly agree	71	21.1
Agree	67	19.9
Agree to some extent	115	34.2
Disagree	69	20.5
Strongly disagree	14	4.2
Do you think that the fear of labor pain was the primary motive for requesting for an epidural injection?		
Strongly agree	121	36.0
Agree	74	22.0
Agree to some extent	59	17.6
Disagree	54	16.1
Strongly disagree	28	8.3
Did a previous negative experience influence your decision not to request for an epidural injection in the future?		
Strongly agree	50	14.9
Agree	44	13.1
Agree to some extent	53	15.8
Disagree	110	32.7
Strongly disagree	79	23.5
Total	336	100.0

**Figure 1:**
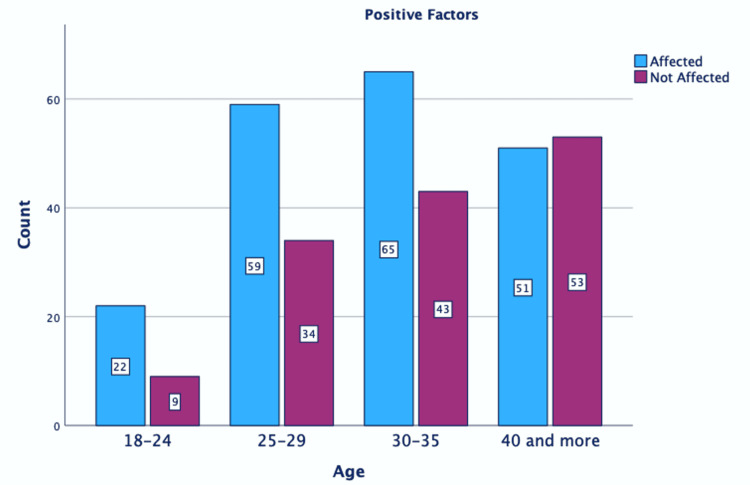
Correlation between positive effects and age. P = 0.76.

**Figure 2:**
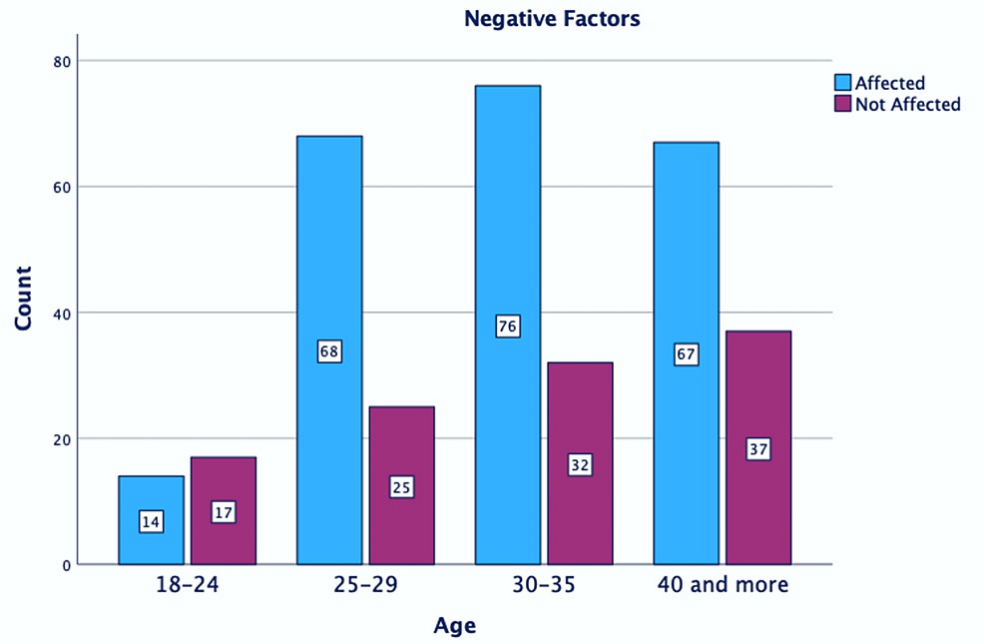
Correlation between negative effects and age. P = 0.28.

Figure 1 and Figure 2 show that participants aged 18-24 years were significantly influenced by the positive factors and not as much by the negative factors.

Our study consisted mainly of women with tertiary education (68.5%), followed by those with secondary education (28%). Positive and negative factors had a low correlation with the educational level and decision-making on whether to use EA. Positive factors had little impact on women with elementary education, as only 0.9% of them stated that positive factors played the greatest role in making the decision (Table 4).

**Table 4:** The impact of education level. Positive factors: P = 0.21. Negative factors: P = 0.001.

Positive factors		Affected	Not affected	Total
Education level	Illiterate	6	0	6
		1.8%	0.0%	1.8%
	Primary school	3	3	6
		0.9%	0.9%	1.8%
	Middle school	54	40	94
		16.1%	11.9%	28.0%
	High school	134	96	230
		39.9%	28.6%	68.5%
Negative factors		Affected	Not affected	Total
Education level	Illiterate	0	6	6
		0.0%	1.8%	1.8%
	Primary school	4	2	6
		1.2%	0.6%	1.8%
	Middle school	57	37	94
		17.0%	11.0%	28.0%
	High school	164	66	230
		48.8%	19.6%	68.5%
	Total	225	111	336
		67.0%	33.0%	100.0%

Previous experience had a significant influence. Of the women who had experienced three or more pregnancies (54.8%), 35.4% indicated that they were more influenced by negative factors, while 30.4% emphasized the impact of positive factors. Women with a single previous pregnancy (20.2%) had been influenced nearly equally by both factors, with a slight difference of approximately 0.3%; 13.4% favored positive factors, whereas 13.1% favored negative factors. Although women who had experienced two pregnancies (25.0%) were considered borderline, 18.5% selected negative factors as the primary determinant of their decision (Table 5).

**Table 5:** The effect of previous pregnancy. Positive factors: P = 0.24. Negative factors: P = 0.30.

Positive factors		Affected	Not affected	Total
Pregnancies	One	45	23	68
		13.4%	6.8%	20.2%
	Two	51	33	84
		15.2%	9.8%	25.0%
	Three or more	101	83	184
		30.1%	24.7%	54.8%
Negative factors		Affected	Not affected	Total
Pregnancies	One	44	24	68
		13.1%	7.1%	20.2%
	Two	62	22	84
		18.5%	6.5%	25.0%
	Three or more	119	65	184
		35.4%	19.3%	54.8%
	Total	225	111	336
		67.0%	33.0%	100.0%

The monthly income of the majority of the participants (31.5%) in this study ranged between 10,000 and 20,000 SAR. There was no significant correlation with positive factors; instead, they were more influenced by the negative factors. Women in the second most common income range, 5,000 to 10,000 SAR, also chose negative factors as strongly influencing their decisions (Table 6).

**Table 6:** The effect of monthly income. Positive factors: P = 0.85. Negative factors: P = 0.01.

Positive factors		Affected	Not affected	Total
Monthly salary	Less than 5,000	53	40	93
		15.8%	11.9%	27.7%
	5,000 to 10,000	60	46	106
		17.9%	13.7%	31.5%
	1,000 to 20,000	67	41	108
		19.9%	12.2%	32.1%
	More than 20,000	17	12	29
		5.1%	3.6%	8.6%
Negative factors		Affected	Not affected	Total
Monthly salary	Less than 5,000	50	43	93
		14.9%	12.8%	27.7%
	5,000 to 10,000	79	27	106
		23.5%	8.0%	31.5%
	1,000 to 20,000	76	32	108
		22.6%	9.5%	32.1%
	More than 20,000	20	9	29
		6.0%	2.7%	8.6%
	Total	225	111	336
		67.0%	33.0%	100.0%

Table 7 shows that both positive and negative factors had an influence on all women, whether married, divorced, or widowed; however, the negative factors (p = 0.10) had a greater influence. Approximately 67% of women thought that negative factors play a major role in the decision-making on whether to use EA. Only 59% of participants thought that positive factors (p = 0.001) had some role in their decision-making.

**Table 7:** The effect of social status. Positive factors: P = 0.001. Negative factors: P = 0.1.

Positive factors		Affected	Not affected	Total
Social status	Married	179	109	288
		53.3%	32.4%	85.7%
	Divorced	10	26	36
		3.0%	7.7%	10.7%
	Widowed	8	4	12
		2.4%	1.2%	3.6%
Negative factors		Affected	Not affected	Total
Social status	Married	193	95	288
		57.4%	28.3%	85.7%
	Divorced	27	9	36
		8.0%	2.7%	10.7%
	Widowed	5	7	12
		1.5%	2.1%	3.6%
	Total	225	111	336
		67.0%	33.0%	100.0%

## Discussion

Our study aimed to understand women’s perspectives on EA in Riyadh, the Saudi Arabian metropolis, and clarify the aspects influencing their decision-making. The study also aimed to dispel common misconceptions about the use of epidural anesthesia and identify the factors that make certain people avoid or request EA. In this study, 336 Saudi women were recruited. Our findings showed that the participants varied in the number of pregnancies, with 55% having had three or more pregnancies. This value was a little less than another study conducted in Saudi Arabia [9]. According to their findings, 57.1% of women had more than three pregnancies. Few received epidural anesthesia during a prior pregnancy, and the majority (79%) had been previously exposed to epidural anesthetic.

Due to a lack of knowledge about the availability of analgesic labor support techniques, women continue to experience excruciating labor pain. Our research showed that more than half of the participants (51%) who learned about epidural anesthesia through family and friends did so because they considered them to be the most reliable sources. In comparison with the results of recently published studies, this proportion was low. A previous study revealed that 67% of Palestinian women became aware of EA usage through friends and relatives [1]. However, another study [10] reported that friends and family members were the primary sources of knowledge about obstetric labor analgesia. A sizable number of women worldwide are unaware of the techniques for giving birth without pain. However, our research revealed that the primary reason for utilizing epidural anesthesia was the fear of pain. The average awareness score of a previous study [5] was 3.66.

A survey [11] revealed that a majority of women (62.5%) had low levels of understanding of EA. This knowledge can determine one’s awareness of the problems associated with EA and the future desire to use it again. A previous study [6] reported that inadequate resources and insufficient general awareness among pregnant women about the function of EA are the main reasons for low patient demand for this type of pain relief during delivery.

A Saudi Arabian study showed that women of childbearing age generally possessed awareness of EA; however, 62.5% of these women had limited comprehension of EA, and most among this group had scant knowledge of the benefits and complications of EA [2]. Similar findings were observed in a study focusing on Saudi women, where the average knowledge level was 45.9%. This study highlighted that despite existing misconceptions and limited awareness and information about EA, there is a considerable desire to adopt EA for alleviating labor pain [12]. Another recent survey conducted in Saudi Arabia found that 16.2% of respondents had become acquainted with EA through various sources, while 60% had only a basic understanding of it. Pregnant women exhibited a lack of awareness about EA, and educational programs effectively increased participant willingness to consider EA as a source of pain relief during labor [7].

However, when the women were educated about EA usage during labor, this raised awareness for the majority of them. In our hospital, women are comfortable using EA to manage labor pain. A good proportion of women of reproductive age are not well-informed on the advantages and risks of EA [13]. It is crucial to educate all pregnant women about EA during antenatal appointments. Information should be provided to all women during antenatal visits either by the obstetrician or anesthetist or through flyers and brochures [2]. In a previous study [14], most pregnant women were aware of and had a positive perception and desire to use EA for labor pain.

On the other hand, pregnant women who lack awareness and understanding of EA might experience heightened labor pain, unfavorably impacting their birth experience. A comprehensive care program for pregnant women should include education about various analgesic methods, with a particular focus on EA [15]. However, the results of our studies in Saudi Arabia confirm that there is a good amount of awareness and knowledge about EA among women. Women have indicated that their knowledge largely stems from family members, relatives, friends, and previous experiences [16].

Certain factors contribute to women’s decision to use epidural anesthesia during childbirth. Our study revealed that 40% of respondents valued a good training program for epidural anesthesia. This result was consistent with that of a previous study [17], which emphasized that health education on EA is a crucial factor that increases the inclination of primigravid women to opt for EA. Therefore, integrating EA education into antenatal care is essential to enhance decision-making regarding the use of EA during labor.

Further, positive experiences of family and friends were mentioned as a factor that may influence a woman’s decision to request EA during labor. Moreover, a good support system is another important factor that may influence women’s decision to request EA. Epidural anesthesia makes labor less laborious and painful; however, it is not risk-free. Our findings revealed that fear of affecting the labor outcome, prior negative experiences, complications, and preexisting medical issues are factors that might negatively influence the decision of women toward EA use.

Our study found that negative factors had a greater influence on women in the largest age group (30-35 years). Negative factors, rather than positive ones, particularly affected women over 40 years of age. Positive factors had the most impact on younger women. All women irrespective of their marital status were affected by both positive and negative factors. Most women stated both that negative factors played a significant role in their choice of epidural anesthesia and that positive factors also played a substantial role in the decision-making process. Our findings also showed that the women’s educational background did not correlate with the influence of either positive or negative factors on the decision-making process regarding EA. However, a favorable disposition and a higher level of education emerged as significant factors in the application of labor pain management techniques [3].

Limitations

Women with lower levels of education were slightly affected by positive factors, which had the greatest role in their decision-making. Previous experiences played a major role, which was also enhanced by positive factors. Moreover, negative factors were not significantly correlated with income. Many factors can cause pain to be perceived differently. In addition, all regions of Saudi Arabia should be studied regarding EA to allow generalizability of the results to the Saudi population. The sample size was insufficient for a multivariate analysis.

## Conclusions

The primary reason for using epidural anesthesia is to alleviate the fear of pain. Addressing knowledge gaps through healthcare practitioners can rectify negative perceptions about EA. Saudi women often seek information about EA from their families and friends due to critical issues and religious considerations. While some Saudi women exhibit good practice of EA, there is limited awareness about it among others. Therefore, efforts should focus on enhancing awareness about EA’s role as a pain reliever and its costs, as it is available in most obstetrics clinics. Implementing health education programs on EA could improve women’s understanding and intention to use it, especially during antenatal care, given the prevalence of multiple pregnancies among Saudi women. However, there is a significant gap between acquiring knowledge and effectively translating it into practice.
